# Study on Acute Toxicity of Amiodarone New Complexes With Cyclodextrin

**DOI:** 10.3389/fphar.2021.640705

**Published:** 2021-03-04

**Authors:** Cristina Mihaela Ghiciuc, Maytham Razaq Shleghm, Cornelia Vasile, Gladiola Tantaru, Andreea Creteanu, Lacramioara Ochiuz

**Affiliations:** ^1^ Department of Pharmacology, Faculty of Medicine, “Grigore T. Popa” University of Medicine and Pharmacy, Iasi, Romania; ^2^ Department of “Carol Davila” University of Medicine and Pharmacy, Bucharest, Romania; ^3^ Physical Chemistry of Polymers Department, Petru Poni Institute of Macromolecular Chemistry, Iasi, Romania; ^4^ Department of Analytical Chemistry, Faculty of Pharmacy, “Grigore T. Popa” University of Medicine and Pharmacy, Iasi, Romania; ^5^ Department of Pharmaceutical Technology, Faculty of Pharmacy, Grigore T. Popa University of Medicine and Pharmacy of Iasi, Iasi, Romania

**Keywords:** amiodarone, cyclodextrin complex, acute toxicity, laboratory animals, new complexes

## Abstract

Amiodarone low solubility and high permeability is the limiting step for its bioavailability, therefore new formulations are needed to improve the solubility of amiodarone either to increase its oral bioavailability or to reduce its toxic effects. Complexation of amiodarone with cyclodextrin results in improved dissolution rate, solubility, and allows for a more controlled drug release. We characterized the acute toxicity of a new amiodarone 2-hydroxypropyl-β-cyclodextrin complex (AMD/HP-β-CD) as powdered form and as a matrix based on Kollidon® and chitosan, administered intraperitoneally in laboratory animals. There were developed two formulations of matrix: one containing only pure AMD as a control sample (Fc) and one containing the inclusion complex with the optimal solubility (F). AMD was equitoxic with HP-β-CD after intraperitoneal administration (289.4 mg/kg for AMD and 298.3 mg/kg for AMD/HP-β-CD), with corresponding histopathological changes. The matrix based formulations presented higher LD50 values for acute toxicity, of 347.5 mg/kg for Fc and 455.6 mg/kg for F10, conducting to the idea of a safer administration because KOL and CHT matrix modified the solubility and controlled the AMD release. The LD50 is 1.5 higher for AMD/HP-β-CD included in a KOL and CHT based matrix compared to the pure AMD, administered intraperitoneally.

## Introduction

Many generic formulations of Amiodarone hydrochloride (AMD) are available for the clinical practice, but there is a high inter-individual variability for AMD (Atanasova and Terziivanov, 2001). There is a need to develop and implement new delivery systems, which combines safety and efficacy, to improve the solubility of amiodarone either to increase its oral bioavailability or to reduce its toxic effects, or intended to shorten the onset of AMD action. AMD is a Class II drug (according to the Biopharmaceutical Classification System) because it has low solubility and high membrane permeability, therefore dissolution in gastrointestinal fluids is the limiting step for its oral bioavailability (Amidon et al., 1995; Benet, 2005). The solubility of a drug influence the choice of formulation for oral or parenteral administration, dissolution, and absorption from the digestive tube, therefore, development of new formulations with high solubility for low solubility compounds is a challenge.

The hydroxypropyl-β-cyclodextrin (HP-β-CD) is a cyclodextrin with very good solubility and lowest toxicity, used to increase the solubility of various poorly soluble drugs (Gould and Scott, 2005; Jacob and Nair, 2018; Muankaew and Loftsson, 2018). Cyclodextrins (CD) are a family of cyclic oligosaccharides with a hydrophobic cavity containing the active compound which is internalized forming an inclusion complex with the drug. Complexation of a drug with cyclodextrins has numerous advantages such as improved dissolution rate, improved bioavailability, safety, and stability and allows for a more controlled drug release (Szejtli, 2005; Tiwari et al., 2010). Thus, enhanced solubility and dissolution rate was obtained *in vitro* in the case of amiodarone complexation with β-cyclodextrin (Riekes et al., 2010). A recent *in vitro* study (Rubim et al., 2017) evaluated the influence of the CD type (β-cyclodextrin, methyl-β-cyclodextrin, and 2-hydroxypropyl-β-cyclodextrin) on the complexation with AMD and on dissolution rate and found enhanced solubility and dissolution rates for inclusion complexes (Rubim et al., 2017). Only a few studies reported *in vitro* characterization of inclusion complexes of HP-β-CD with amiodarone (Rubim et al., 2017; Creteanu et al., 2019), but there are no data available about the acute toxicity of these systems.

Complexation of AMD with HP-β-CD (AMD/HP-β-CD) increases its solubility and bioavailability without any modification of its structure (Păduraru et al., 2013; Creteanu et al., 2016a; Creţeanu et al., 2016b; Creteanu et al., 2017), therefore also the acute toxicity should increase. In one of our previous papers (Creteanu et al., 2019), the physicochemical properties have been evaluated for the inclusion complex formation with AMD and for two new formulations with a matrix based on Kollidon® (KOL) and chitosan (CHT), and it was established a considerably increase of the dissolution rate of AMD from the inclusion complexes, compared to dissolution of the pure AMD. The aim of this study was to evaluate the acute toxicity of AMD from new complexes with cyclodextrin AMD/HP-β-CD as powdered form and as a matrix based KOL and CHT, administered intraperitoneally in laboratory animals. There were developed two matrix based inclusion complex formulations: one containing only pure AMD as a control sample (Fc) and one containing the inclusion complex with the optimal solubility (F10).

## Materials and Methods

### Materials

Amiodarone (AMD) (Mw = 645.32 Da) of 99.85% purity have been obtained from Zhejiang Sanmen Hengkang Pharmaceutical Co. Ltd., China. HP-β-CD of 99.70% purity have been obtained from Roquette, France. Polyoxyethylene (Hodge and Sterner, 1949) sorbitan monooleate (polysorbate 80, Tween 80) have been obtained from Sigma-Aldrich, Inc. Kollidon^®^ SR (KOL), a mixture constituted from 80% poly(vinyl acetate) and 20% polyvinylpyrrolidone (povidone), and chitosan (CHT) were purchased from BASF, Germany.

AMD/HP-β-CD contains AMD:cyclodextrin in ratio 1:1. The substances were administered in constant volume (0.1 ml/10 g b.w.) as freshly prepared suspension with distilled water and 1% Tween-80. The solutions for acute toxicity study were prepared using the same weight of powder of AMD, respectively of AMD/HP-β-CD, Fc and F10 powders.

### Experimental Animals

The protocol of the experimental study was approved by the Institutional Ethics Committee of “Grigore T. Popa” University of Medicine and Pharmacy of Iasi, Romania (No. 23983/2014). Healthy nulliparous and non-pregnant female Balb/C mice (weighting 20–25 g) were purchased from Cantacuzino Institute (Bucharest, Romania). The animal study was done in accordance with the international guidelines (National Research Council US Committee for the Update of the Guide for the Care and Use of Laboratory Animals, 2011). The animals were housed in plastic cages with stainless steel mesh lids in a ventilated room with standard environmental conditions: 12 h light-dark cycle, room temperature 24 ± 2°C and 50–70% relative humidity. They were provided *ad libitum* with standard rodent pellet food and tap water, for 5 days before testing.

### Acute Toxicity Testing

To obtain the 50% lethal dose (LD50) of the AMD, respectively AMD/HP-β-CD, the experiments were designed in accordance with the “Up and Down Procedure” (UDP) provided by the Organization for Economic Cooperation and Development (OECD) Guideline 425 (OECD, 2008) and described by Dixon (Dixon, 1965). Limit test was performed at 2,000 mg/kg p.o. as single dose administered according to body weight. Main test was performed with doses adjusted by a constant multiplicative factor (namely 1.6) for this experiment: 175, 275, 440, 690 and 1,090 mg/kg b.w. The animals were fasted for 12 h prior to dosing and the single dose for each successive animal was adjusted up or down depending on the previous outcome, after 24 h one by one. Animals were closely observed during the first 30 min, then hourly for the next 6 h, at 24 h and daily for the next 14 days to record mortality and all the relevant clinical symptoms of acute toxicity (evaluation of skin and fur, grooming, posture and gait, salivation, tremor, convulsion, hyperactivity, apathy, respiratory depression, and coma). The LD50 is calculated using the maximum likelihood method with a sigma of 0.5 (OECD, 2008).

### Histopathological Study

The vital organs (liver, kidney, lungs and heart) isolated from the dead mice were preserved in 10% formalin, then embedded in paraffin wax. Paraffin sections (5 mm) were stained with hematoxylin and eosin (HE) to be studied under a light microscope for morphological alterations.

## Results

### Acute Toxicity of AMD, of AMD/HP-β-CD, of AMD Control Matrix (Fc), and of AMD/HP-β-CD Fatrix (F)

#### Vital Signs

During the entire study period, no unusual clinical signs were observed in the mice that received 175 mg/kg b.w of AMD, respectively AMD/HP-β-CD, Fc, and F. Supplementary Table S1 summarizes some behavioral responses of mice from the first 30 min and hourly during the first 6 h after administration of a single dose of administered substances. A decrease in sensitivity and activity, tremor and convulsions were observed at 690 mg/kg b.w., during the first hour, followed by death in the case of AMD, respectively AMD/HP-β-CD. A few animals (3 out of 4) showed slight symptoms after the dose of 275 mg/kg for AMD/HP-β-CD and died 6 h after the administration of the substance. For matrix based inclusion complexes, a decrease in sensitivity and activity, tremor and convulsions were observed at 1,090 mg/kg b.w., during the first hour, followed by death for Fc and F10.

LD50 values of AMD, respectively AMD/HP-β-CD, using up and down procedure were determined to be 289.4 mg/kg for AMD and 298.3 mg/kg for AMD/HP-β-CD. For the matrix based formulations, LD50 values were 347.5 mg/kg for Fc and 455.6 mg/kg for F10.

### Histopathological Evaluation

**FIGURE 1:**
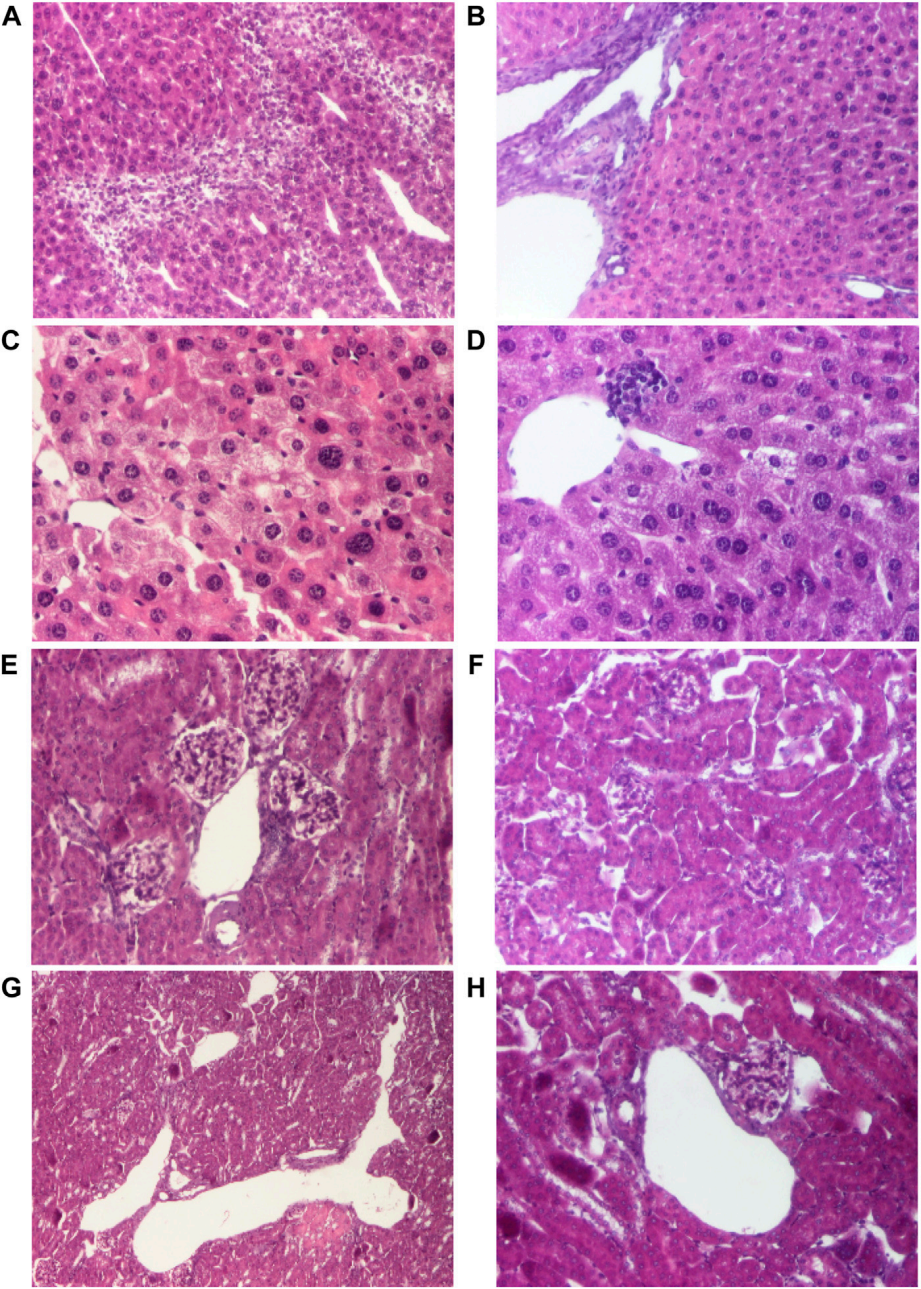
Histological changes of the main organs in the dead mice after doses of 690 mg/kg of AMD **(A, C, E, G)** compared to 440 mg/kg of AMD **(B, D, F, H)**. Representative pictures from HE staining sections of the liver with hepatitis aspect (hematoxylin-eosin HE stain, ×100) **(A)**, liver–portal space (hematoxylin-eosin HE stain, ×100) **(B)**, degeneration of hepatocytes (hematoxylin-eosin HE stain, ×200) **(D)**, hepatocytes (hematoxylin-eosin HE stain, ×200) **(D)**, renal cortex and arteriole (hematoxylin-eosin HE stain, ×200) **(E)**, renal cortex (hematoxylin-eosin HE stain, ×100) **(F)**, renal cortex and dilated renal veins (hematoxylin-eosin HE stain, ×40) **(G)**, renal cortex and dilated renal veins (hematoxylin-eosin HE stain, ×100) **(H)**

**FIGURE 2:**
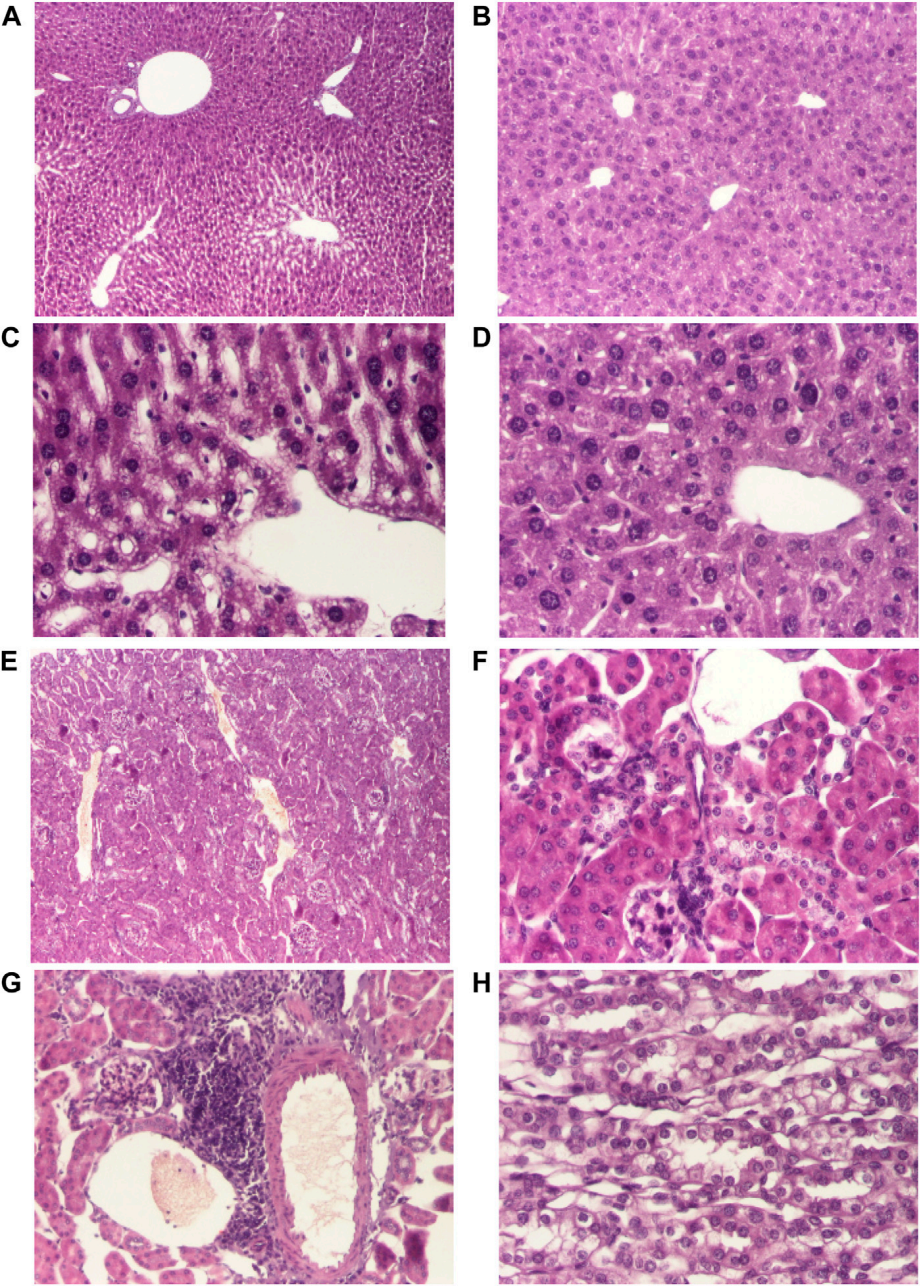
Histological changes of the main organs in the dead mice after doses of 690 mg/kg of AMD/HP-β-CD **(A, C, E, G)** compared to 440 mg/kg of AMD/HP-β-CD **(B, D, F, H)**. Representative pictures from HE staining sections of the liver (hematoxylin-eosin HE stain, ×40) **(A, B)**, hepatocytes (hematoxylin-eosin HE stain, ×200) **(C, D)**, renal cortex (hematoxylin-eosin HE stain, ×40) **(E)**, renal cortex (hematoxylin-eosin HE stain, ×100) **(F)**, interstitial nephritis (hematoxylin-eosin HE stain, ×100) **(G)**, renal medulla (hematoxylin-eosin HE stain, ×200) **(H)**

Necropsy was carried out in all animals immediately after death. Internal organs (liver, kidney, lungs and heart) were examined for macroscopic alterations induced by AMD, respectively AMD/HP-β-CD, and collected to perform histopathological analysis (Figures 1, 2). No macroscopic changes were observed.

Histopathological examination of liver and kidney fragments in animals receiving toxic doses of 690 mg/kg of AMD revealed suggestive aspects of drug-induced hepatotoxicity (Figures 1A,C), compared to doses of 440 mg/kg of AMD (Figures 1B,D). In the kidney, there are typical changes induced by 690 mg/kg of AMD (Figures 1E,G) compared to doses of 440 mg/kg of AMD (Figures 1F,H).

Histopathological examination of liver fragments in animals receiving toxic doses of 690 mg/kg of AMD/HP-β-CD revealed suggestive aspects of drug-induced hepatotoxicity (Figures 2A,C), compared to doses of 440 mg/kg of AMD/HP-β-CD (Figures 2A,B). Histopathological examination at the renal level revealed few changes characteristic of nephrotoxicity induced by 690 mg/kg of AMD/HP-β-CD (Figures 2C,D).

**FIGURE 3:**
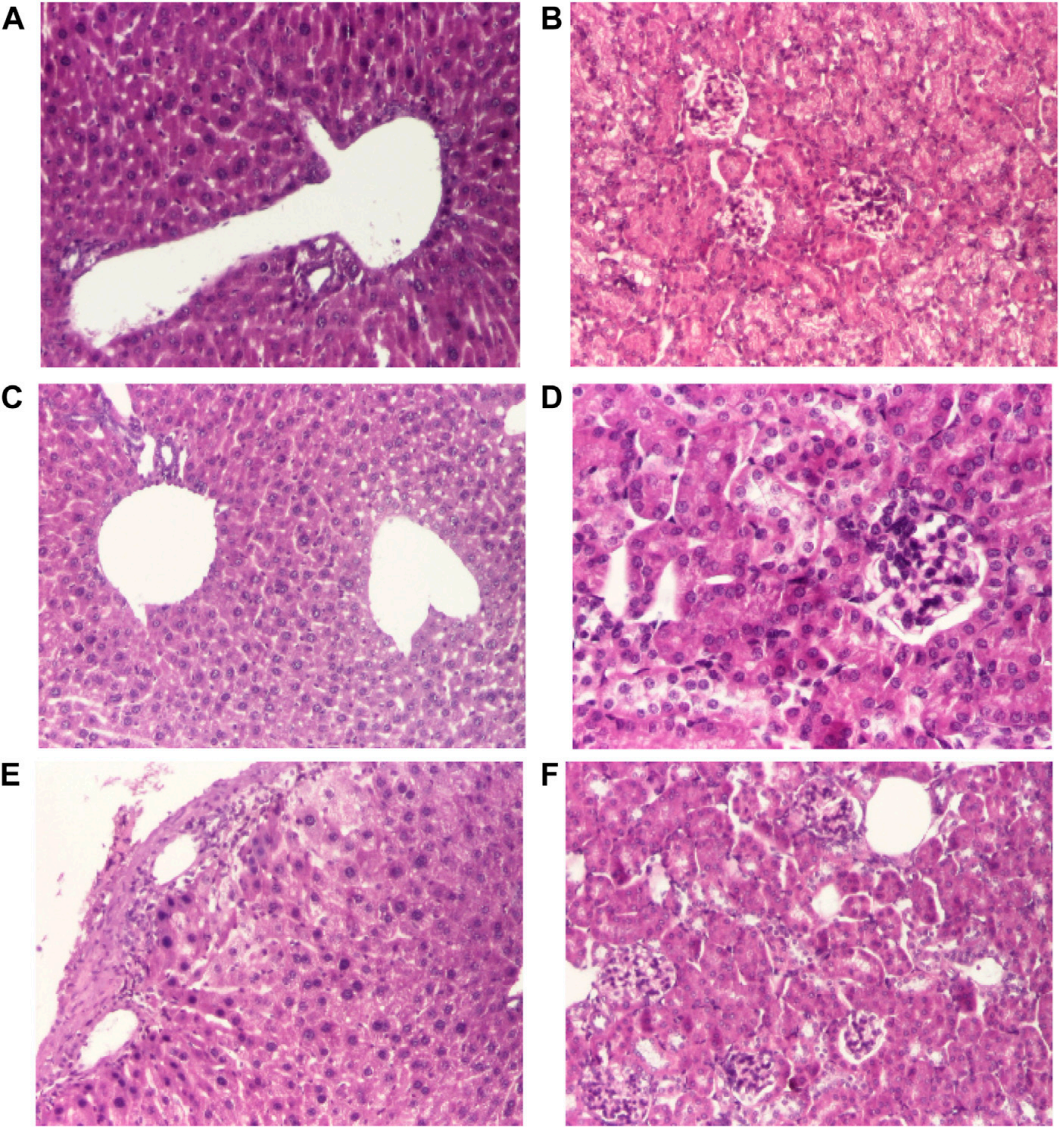
Histological changes of the main organs in the dead mice after doses of 690 mg/kg of Fc **(A, B)** and F10 **(C, D)** and after doses of 1,090 mg/kg of F10 **(E, F)**. Representative pictures from HE staining sections of the liver (hematoxylin-eosin HE stain, ×100) **(A, C, E)**, renal (hematoxylin-eosin HE stain, ×100) **(C, D, F)**

Histopathological examination of liver and kidney fragments in animals receiving toxic doses of 690 mg/kg of Fc (Figures 3A,B) and F10 (Figures 3C,D) few changes characteristic of hepatic and nephrotoxicity. There were analyzed only for the doses of 1,090 mg/kg for F10 which revealed signs of hepatic and nephrotoxicity (Figures 3E,F).

## Discussion

Our study found that AMD/HP-β-CD has similar acute toxicity with pure AMD after oral administration in mice: 289.4 mg/kg for AMD and 298.3 mg/kg for AMD/HP-β-CD. Similar doses of these compounds produced equivalent degree of acute toxicity only for AMD, respectively AMD/HP-β-CD. For the matrix based formulations, LD50 values were 347.5 mg/kg for Fc and 455.6 mg/kg for F10. The KOL and CHT matrix induced high differences in the solubility and controlled AMD release: The LD50 is 1.5 higher when AMD is complexed with HP-β-CD and included in F10 matrix than for pure AMD. These formulations are considered moderately toxic (dose between 50–500 mg/kg), according to Hodge and Sterner scale for the evaluation of toxicity with the help of LD50 (Hodge and Sterner, 1949), with F10 being closer to the limit of slightly toxic. Amiodarone is difficult to administer because of its narrow toxic-therapeutic range (Tamargo et al., 2015). In our study, intraperitoneally administration of AMD had comparable results to other acute toxicity studies with Amiodarone: LD50 of 294.0 mg/kg body weight in female mice after intravenous administration (Barle et al., 2013). The matrix based formulation increased the solubility and modified the release of the substance from the formulation.

Hydroxypropyl-β-Cyclodextrin (HP-β-CD) is highly biocompatible and pharmacologically inactive, therefore it was considered a safe material to improve the solubility and the bioavailability of AMD (Patel and Hirlekar, 2019). A cyclodextrin-complex formulation containing a well-known long used drug is considered a “super generic”, superior in its performance when compared to other products which contain the same known active substance (Szejtli, 2005). In the case of oral administration, pharmacokinetic studies showed that drug/cyclodextrin complexes have shorter Tmax, higher Cmax and larger AUC values as a result of the increased bioavailability (Szejtli, 2005). Pharmacodynamic studies showed that most drug/cyclodextrin complexes from the market belong to these super generic drugs having a higher and quicker therapeutic effect (Szejtli, 2005). A recent pharmacokinetic study conducted in rats after intravenous bolus administration showed that the inclusion of HP-β-CD in the solution of the administered substance improved blood compatibility (Mantik et al., 2019). Polysorbate 80 (Tween 80), a nonionic surfactant, is considered non-toxic, being used as an additive, as emulsifier, as dispersant, or as stabilizer in various type of foods, pharmaceutical preparations, or cosmetics (National Toxicology Program, 1992; EFSA FEEDAP Panel, 2016).

On the other hand, Amiodarone has a narrow therapeutic ratio and it is considered a critical dose drug, therefore, increasing the solubility and the controlled AMD release to reduce the active drug dose in the new super generic cyclodextrin formulation was considered an obvious idea. In our experiment, LD50, based on up and down procedure, was 289.4 mg/kg for AMD, similar to LD50 values from literature (Barle et al., 2013) and 298.3 mg/kg for the new inclusion cyclodextrine complex HP-β-CD, meaning that AMD and its complex, HP-β-CD, had equivalent degree of toxicity. Single dose administration of 440 mg/kg and of 690 mg/kg of the classical AMD or of our new formulation HP-β-CD produced equivalent toxic effects in mice: both caused complex symptoms such as changes in breathing, agitation, increased heart rhythm, convulsions, tremors, cyanosis, falls, defecation, urination, and piloerection. In mice, both AMD and HP-β-CD, and matrix formulations determined hepatic and renal toxicity lesions, similar to those reported in the literature after high doses of AMD. These effects were not seen in matrix formulations Fc and F10. In humans, AMD-induced acute toxicity syndrome is unusual, manifested with acute liver and renal failure (Paudel et al., 2016). Central nervous system manifestations were described in the literature when toxic doses of AMD were administered. In the literature, cases of pulmonary damage, e.g., acute pulmonary edema, have been reported following administration of toxic doses of AMD in humans (Kaya et al., 2017).

## Conclusion

Our single dose acute toxicity study showed that the same amount of AMD was equitoxic with HP-β-CD, after intraperitoneal administration, the lethal doses (DL50) of these pharmaceutical forms included them into the category 3 of toxicity (moderately toxic substances). For the matrix based formulations, LD50 were higher conducting to the idea of a safer administration because KOL and CHT matrix induced high differences in the solubility and controlled AMD release. The LD50 is 1.5 higher for AMD/HP-β-CD included in a KOL and CHT based matrix compared to the pure AMD, administered using the same route.

## Data Availability

The original contributions presented in the study are included in the article/Supplementary Material, further inquiries can be directed to the corresponding authors.
