# Supplementary material for: Study on Acute Toxicity of Amiodarone New Complexes With Cyclodextrin
Source: Front Pharmacol. 2021 Mar 4;12:640705. doi: 10.3389/fphar.2021.640705 (PMC8058604; doi:10.3389/fphar.2021.640705)
Supplement: Supplementary file 1 [file DataSheet1.pdf]

Table Clinical signs of acute toxicity in animals treated with the same doses.

Dose (mg/kg) (n= deaths/survivals)	AMD				AMD/HP-β-CD				Fc				F10			
	175 mg/kg (n= 2/2)	275 mg/kg (n= 3/3)	440 mg/kg (n= 1/2)	690 mg/kg (n= 1/1)	175 mg/kg (n= 2/2)	275 mg/kg (n= 3/4)	440 mg/kg (n= 2/3)	690 mg/kg (n= 1/1)	275 mg/kg (n= 0/2)	440 mg/kg (n= 3/2)	690 mg/kg (n= 1/2)	1090 mg/kg (n= 1/0)	275 mg/kg (n= 2/2)	440 mg/kg (n= 3/4)	690 mg/kg (n= 2/3)	1090 mg/kg (n= 1/1)
skin and fur	N	N	+	+	N	+	++	+	N	N	+	+	N	+	++	+
eyes	N	N	N	+	N	N	+	+	N	N	N	+	N	N	+	+
grooming	N	N	+	+	N	+	+	+	N	N	+	+	N	+	+	+
posture	N	N	+	+	N	+	+	+	N	N	+	+	N	+	+	+
gait	N	N	+	+	N	+	+	+	N	N	+	+	N	+	+	+
salivation	N	N	N	+	N	N	+	+	N	N	N	+	N	N	+	+
tremor	-	-	+	+	-	+	+	+	-		-	+	-	+	+	+
convulsion	-	-	-	++	-	-	-	++	-		-	++	-	-	-	++
hyperactivity	-	-	-	-	-	-	-	-	-		-	++	-	-	-	++
apathy	-	-	-	+	-	-	-	+	-		-	+	-	-	-	+
tachycardia	-	-	++	++	-	-	++	++	-		-	++	-	-	+	++
respiratory depression	-	-	+++	+++	-	+	+++	+++	-		+	+++	-	-	++	+++
coma	-	-	-	+	-	-	-	+	-		-	+	-	-	-	-

Legend: N Normal, - No sign observed, + Slight symptoms observed, ++ Moderate symptoms observed, +++ Severe symptoms observed.
